# Laboratory Findings in Typhoid

**Published:** 1911-07

**Authors:** J. Edgar Paullin

**Affiliations:** Pathologist to Ga. State, Board of Health


### LABORATORY FINDINGS IN TYPHOID.

J. Edgar Paullin, B.A., M.D.,

*Pathologist to Ga. State, Board of Health.*

The results given by laboratory investigations in this particular infection furnished exceedingly important evidence in enabling the clinician to diagnosticate early the affection and in furnishing information of great importance should complications arise. These methods are not used as frequently as they should be, and as a result their importance is underestimated by the majority of practitioners.

Regarding the blood picture; in general, there is very little change in the number of the red blood cells during the first two weeks of the infection. About the end of the third week there is noted a slight diminution in the number of the red cells, varying from 400 to 1500 thousand cells to the cubic mm. of blood. Shortly after the establishment of convalescence the cells gradually return to normal. Sweats, diarrhoea and vomiting during the course of the disease cause at times an apparent increase in their number. After several hemorrhage they are considerably decreased, although not in as great proportion as the haemoglobin, in fact, it is often possible to diagnose a hemorrhage before the blood appears in the stool by careful estimations of the haemoblobin and red cells during the course of the disease.

The Leucocytes at the beginning of the infection are slightly increased in number. After the disease has progressed for a week or ten days the count drops as a rule to 4000 or 5000 cells per cm. and they remain at about this level throughout the course of an uncomplicated case of typhoid. In certain cases we frequently find leucocyte counts as low as 2000; these as a rule are severe cases in which the symptoms of toxemia are marked. I would call attention to the fact that baths increase temporarily the number of leucocytes, this increase lasts for 1½ to 3 hours after the bath. The normal relative proportion of the leucocytes is considerably altered during the infection. The monynuclears are increased in number, while there is a decided decrease in the number of polymorphynuclears. The normal number of polymorphynuclears is from 68 to 70 per cent, whereas in typhoid fever they will frequently drop to 50 or 58 per cent. The monynuclears increase in practically the same percentage as the polymorphynuclears diminish.

The importance of a slight leucocytosis during the course of the disease cannot be overestimated. There are four possible causes to explain this increase: 1st, septic processes, such as the development of furuncles; 2nd, inflammatory processes, such as cholecystitis, phlebitis, parotitis and pneumonia; 3rd, following hemorrhage; there is a slight leucocytosis which reaches its maximum in 24 hours; 4th, perforation of the intestine. Regarding this condition an entirely erroneous idea has been expressed by some concerning the behavior of the leucocytes. Under ordinary conditions the onset of a peritonitis is heralded by an increase in the number of the leucocytes and generally speaking this is true with cases of typhoid fever, but the leucocytes generally are not increased in such a manner as one would naturally expect. Frequently the increase will only amount to 600 or 800 cells per cu. mm., and occasionally it happens that perforation takes place with no discoverable change in the blood. Instances have been recorded of cases where perforation was accompanied by a decrease in numberof the leucocytes. When there is no discoverable change in the relative number of the leucocytes under this condition, there is nearly always a change in the relative proportion of the cells as they occur in the blood; so that it is necessary not only to enumerate the leucocytes, but also to determine the relative percentage ot the cells as they occur in the blood by making a differential count of 1000 cells.

When one suspects that a perforation has taken place, a leucocyte count should be made at once, together with a differential count of 1000 cells. The count should then be made every hour thereafter to determine the slightest change that will be evident in the blood. With the clinical evidence pointing to a possible perforation a rise of 600 or 800 cells in the course of three or four hours and an increase in the percentage of polymorphoneuclears would almost make it certain that perforation had occurred.

The Haemoglobin itself follows fairly closely the course of the red cells; that is, it is diminished during the third week of the illness and gradually inreases to normal during convalescence. After hemorrhage it is decreased more than the relative number of the red cells would indicate.

### Coagulation Time.

It is also necessary that a clinician should have knowledge of the coagulation time of the patient's blood, as soon as typhoid fever is diagnosed. It is a very simple and easy thing to test the blood to determine this fact, and I firmly believe that serious and probably fatal hemorrhages have been prevented by knowing this in time to fortify the patient against a possible severe and fatal hemorrhage by increasing this coagulation. The apparatus for estimating this I now pass about for your inspection.

### Blood Cultures.

The information which one gains from blood cultures in a suspicious case frequently enables us to make a positive diagnosis of an otherwise obscure malady. The method which I have used for some time in obtaining the blood consists in sterilizing the patient's arm after the methods used in surgical procedures, putting a turniquet above the elbow and aspirating with a glass syringe from the median vein 5 or 10 cc. of blood. This is then inoculated into several flasks containing boullion to which has been previously added a small amount of a killed culture of the typhoid bacillus. This is known as the method of Cole, Davidson and Cronk. By adding the killed typhoid organisms (which have been previously tested for their sterility) the amboceptor present in the human blood from which the culture is made is destroyed, and any typhoid organism which might be present is allowed to grow more rapidly than it otherwise would. These authors claim that they are able by the use of this method to find the typhoid organism in 70 per cent of the cases examined. For a long time the inoculation of media containing bile was supposed to give the best culture for the growth of this organism. The previous method has given more satisfactory results in my hands than any other that has been tried.

### Widal.

By some the importance of the Widal reaction is underestimated, by others its importance is decidedly overrated. The opinion which I hold concerning its usefulness depends upon two factors: 1st, the method employed in making the test and second, the experience of the individual who does the work. To my mind the Widal reaction which is done with dry blood collected on filter paper, blotting paper or in any other way, is absolutetly worthless, and it is a waste of time to attempt to gain any information by this method. There is no scientific basis or excuse for any such procedure. The elements entering into the reaction are all inaccurate and undetermined. No definite idea is formed or can be formed of the amount of substance which goes into solution when the dried blood is mixed with water, and since this is not known, any result which might be obtained by the use of such a method is absolutely worthless. The correct way of doing the Widal is to obtain the patient's blood in a capillary pipette, allow the blood to coagulate; mix with a definite quantity of 8 per cent. salt solution and use this for testing with the typhoid bacillus. Three dilutions should be made in all cases a 1 :10, 1 :50 and 1 :100. The results should be read after one hour's time nad the interpretation based entirely on this. It is quite true that many cases of typhoid fever do not give a positive Widal until they are practically recovered from the disease. It is also true that the greater number of cases give a positive Widal by the end of the 10th to 14th day of the disease. Should a positive Widal be obtained by the method which I have previously described, one can be certain that the patient has or has had typhoid fever. I would, however, say that because the patient's blood never gives a positive test by this method, it is by no means certain from this that the individual has not the disease.

It has been shown by Bordet and Gengou in working with the blood of typhoid patients that there are present in the blood of these individuals amboceptors which can be discovered by making a complement fixation test on the blood of these patients. This test is of about the same importance as the Widal, and is positive in about the same number of cases.

### Urine.

The urine of these cases shows little of moment; within the first ten days albumin is present in about 66 per cent of the cases, and casts in 58 per cent, the quantity of urine depends upon the amount of water ingested.

The diazo-reaction of Erlich shows very little of diagnostic import, as it is frequently present in malaria, tuberculosis, pneumonia and the various exanthemata. The same is true of the Methylene blue reaction described by Rosso.

The typhoid bacillus may be recovered from the urine, although it is rather difficult if there also exists a cystitis.

### FECES.

The stools are as a rule thin, pea soup, and offensive in odor. Occult blood is usually present after the second week. The isolation of the typhoid bacillus from the stools has been frequently accomplished by the use of a special media described by Conradi and Drigalski. The organisms, however, do not occur in any considerable number in these cases.

